# Theaflavin-3,3′-Digallate Plays a ROS-Mediated Dual Role in Ferroptosis and Apoptosis via the MAPK Pathway in Human Osteosarcoma Cell Lines and Xenografts

**DOI:** 10.1155/2022/8966368

**Published:** 2022-10-25

**Authors:** Tao He, Xiaohong Lin, Chaohua Yang, Zhiyu Chen, Linbang Wang, Qiaochu Li, Jingjin Ma, Fangbiao Zhan, Yang Wang, Jin Yan, Zhengxue Quan

**Affiliations:** ^1^Chongqing Medical University, Chongqing 400016, China; ^2^Department of Orthopedics, The First Affiliated Hospital of Chongqing Medical University, Chongqing 400016, China; ^3^Orthopedic Laboratory of Chongqing Medical University, Chongqing 400016, China; ^4^Department of Orthopaedic Trauma, Chongqing General Hospital, Chongqing 401147, China; ^5^Department of Ultrasound, Chongqing General Hospital, Chongqing 401147, China; ^6^Department of Orthopedics, Chongqing University Three Gorges Hospital, Wanzhou, Chongqing 404000, China; ^7^Department of Neurosurgery, The First Affiliated Hospital of Chongqing Medical University, Chongqing 400016, China

## Abstract

Globally, osteosarcoma (OS) is the most prevalent form of primary bone cancer in children and adolescents. Traditional neoadjuvant chemotherapy regimens have reached a bottleneck; thus, OS survivors have unsatisfactory outcomes. Theaflavin-3,3′-digallate (TF3) exhibits potent anticancer properties against many human cancers. Nevertheless, the biological effects and the underlying molecular mechanism of TF3 in human OS remain unclear. The objective of this study was to investigate the effects of TF3 on human OS cell lines and mouse xenograft models. The results showed that TF3 reduced cell viability, suppressed cell proliferation, and caused G0/G1 cell cycle arrest in both MG63 and HOS cell lines in a concentration-dependent manner. TF3 also altered the homeostatic mechanisms for iron storage in the examined cell lines, resulting in an excess of labile iron. Unsurprisingly, TF3 caused oxidative stress through reduced glutathione (GSH) exhaustion, reactive oxygen species (ROS) accumulation, and the Fenton reaction, which triggered ferroptosis and apoptosis in the cells. TF3 also induced MAPK signalling pathways, including the ERK, JNK, and p38 MAPK pathways. Furthermore, oxidative stress was shown to be the primary reason for TF3-induced proliferation inhibition, programmed cell death, and MAPK pathway activation in vitro. Moreover, TF3 exhibited markedly strong antitumour efficacy in vivo in mouse models. In summary, this study demonstrates that TF3 concomitantly plays dual roles in apoptotic and ferroptotic cell death by triggering the ROS and MAPK signalling pathways in both in vitro and in vivo models.

## 1. Introduction

Osteosarcoma (OS), the most prevalent form of primary bone cancer in children and young adults worldwide, principally occurs in the extremities. Cancerous cells mainly arise from abnormal differentiation of mesenchymal stem cells [1, 2]. Surgery and neoadjuvant chemotherapy are common methods of clinical treatment for patients with OS and have achieved great progress. Nonetheless, traditional neoadjuvant chemotherapy regimens have reached a bottleneck, and no significant changes have been observed in the survival rates for OS patients over the past few years [3]. In addition, OS patients with lung metastases continue to have a poor prognosis [4]. Thus, increased efforts should be made to improve survival by developing novel active agents or new strategies that take advantage of cellular molecular targets [5]. One promising strategy is the screening of anticancer agents found in natural products to use the advantages of phytochemicals to benefit human health.

**Figure 1:**
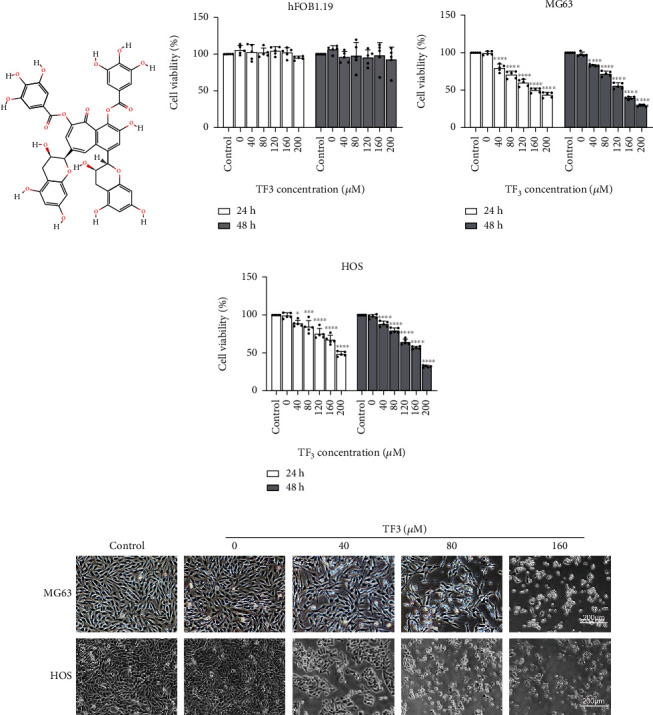
TF3 selectively inhibited cell viability in OS cell lines. OS cells were treated with 0.05% DMSO (vehicle control) or the indicated concentrations (0, 40, 80, 120, 160, or 200 *μ*M) of TF3. (a) Chemical structure of TF3 (retrieved from PubChem). (b–d) CCK-8 assay results showing the viability of HOS, MG-63, and hFOB1.19 cells after treatment for 24 or 48 h. The data are presented as the mean ± SD (*n* = 5). ^∗^*P* < 0.05, ^∗∗^*P* < 0.01, ^∗∗∗^*P* < 0.001, and ^∗∗∗∗^*P* < 0.0001 vs. the vehicle control group. (e) Morphological features of MG-63 and HOS cells observed by microscopy. After 24 h of treatment with the indicated dose of TF3, the cell number was decreased, and the cells exhibited a rounded, shrunken morphology.

Chemopreventive agents are a large group of biologically active compounds found in plants that inhibit the growth, progression, invasion, and metastasis of OS by targeting multiple signalling pathways [6]. Over the past 70 years, approximately half of the clinically used chemotherapy agents in Western countries have been derived or obtained from chemopreventive agents [7]. Natural polyphenols have become important sources for potential new anticancer drugs [8]. Theaflavin-3,3′-digallate (TF3), an active tea polyphenol (Figure 1(a)) and one of the most promising natural polyphenols, has exhibited potent anticancer properties against different types of carcinoma cells [9]. There is some evidence that TF3 inhibits the CaN-NFAT signalling pathway, thereby preventing pathological cardiac hypertrophy (PCH). TF3 may be a natural compound that can prevent PCH and heart failure [10]. In rats, TF3 protected cartilage from degradation and alleviated osteoarthritis (OA), suggesting that TF3 offers potential as a treatment for OA [11]. Furthermore, TF3 regulates genes and signalling pathways involved in reproductive and developmental processes, as well as mTOR, which regulates autophagy, delaying ovarian ageing in aged female mice [12].

Although the biological functions of TF3 in a variety of malignancies have been widely demonstrated, whether TF3 can inhibit human OS and the pathway that mediates anticancer effects remain unknown. We hypothesized that TF3 has an antiosteosarcoma effect. To further expand the use of this natural compound, the molecular mechanism of the antitumour function of TF3 in human OS must be understood. In this study, we aimed to elucidate the antitumour effects and mechanisms through which TF3 acts on human OS.

## 2. Materials and Methods

### 2.1. Materials

TF3 was purchased from Desite Biotechnology Co., Ltd. (Chengdu, China) and was dissolved in DMSO before the experiment. Ferrostatin-1 (Fer-1) was purchased from MedChemExpress (New Jersey, USA). DMSO was purchased from BioFroxx (Einhausen, Germany). Z-VAD-FMK was purchased from Selleck (Selleck, USA). FerroOrange and a Cell Counting Kit-8 (CCK-8) were purchased from Dojindo Molecular Technologies (Kumamoto, Japan). SDS–PAGE gels and 5× Omni-Easy Protein Sample Loading Buffer were purchased from EpiZyme (Shanghai, China). N-acetyl cysteine (NAC), penicillin–streptomycin, a JC-1 assay kit, a DCFH-DA probe, a malondialdehyde (MDA) assay kit, the probe dihydroethidium (DHE), a BeyoClick™ EdU Cell Proliferation Kit with Alexa Fluor 555, Hoechst 33258 dye solution, RIPA lysis buffer, phenylmethylsulfonyl fluoride (PMSF), phosphatase inhibitors, QuickBlock™ Blocking Buffer, a BCA protein assay kit, and bovine serum albumin (BSA) were purchased from Beyotime Biotechnology (Shanghai, China). A GSH assay kit was purchased from Solarbio (Beijing, China). Primary antibodies against p38, phospho-p38, GAPDH, JNK, phospho-JNK, ERK1/2, phospho-ERK1/2, cleaved caspase-3, caspase-3, P21, Ki67, Bax, Bcl-2, and cytochrome C, as well as secondary antibodies, were purchased from Cell Signaling Technology (CST, USA). A rabbit polyclonal antiferritin heavy chain (FTH) antibody (DF6278) was purchased from Affinity Bioscience (China). A TransDetect® In Situ Fluorescein TUNEL Cell Apoptosis Detection Kit, DAB chromogenic solution, Prussian blue solution, and anti-Ki67 were purchased from Servicebio (GB11030, Wuhan, China). Antibodies against GPX4 and JNK were purchased from Abcam Inc. (MA, USA).

### 2.2. Cell Lines

The human OS cell lines MG63 and HOS and hFOB1.19 human osteoblasts were obtained from the American Type Culture Collection (ATCC, USA) and cultured in DMEM (Gibco, Grand Island, NY, USA) containing 10% foetal bovine serum (Wisent, Montreal, Canada) and 100 units/mL penicillin–streptomycin (Beyotime, China) at 37°C in a 5% CO_2_ incubator.

### 2.3. Cell Viability

The two human OS cell lines and hFOB1.19 cells were seeded in 96-well cell culture plates at a density of 3 × 10^3^ cells/well and incubated overnight to allow adherence. Then, the cells were treated with various concentrations of TF3 for 24 or 48 h. The viability of the aforementioned cell lines after TF3 treatment was detected using a CCK-8 assay (Dojindo Molecular Technologies, Kumamoto, Japan). To determine how different inhibitors (the caspase inhibitor z-VAD-FMK (20 *μ*M), ferroptosis inhibitor Fer-1 (2 *μ*M), and antioxidant NAC (2 mM)) affect cell viability after pretreatment, OS cells were treated with TF3 (160 *μ*M) with or without the aforementioned inhibitors for 24 h. Following the different treatments, the cells were incubated for 1 to 2 h at 37°C with a 10% CCK-8 solution in each well. Cell viability was determined by assessing the optical density (OD) values at 450 nm with a microplate reader (Thermo Fisher, Massachusetts, USA).

### 2.4. Transmission Electron Microscopy (TEM)

The different OS cell lines were treated with DMSO (as the control) or TF3 (160 *μ*M) for 24 h. The cells were collected and fixed in 2% paraformaldehyde plus 3% glutaraldehyde for 24 h. Following washing with 0.1 M cacodylate buffer, the cells were postfixed with 2% cacodylate buffered osmium tetroxide at room temperature for 1 h. Next, a series of ethanol concentrations (50% to 100%) was applied to dehydrate the samples. The embedded and sliced samples were subsequently stained with both methanolic uranyl acetate and lead citrate. TEM images were obtained with a JEM-1400 transmission electron microscope (Tokyo, Japan).

### 2.5. Mitochondrial Membrane Potential (MMP) Assay

MG63 and HOS cells were plated on 6-well cell culture plates at 1 × 10^5^ cells/well and incubated to reach adherence. The following day, OS cells were incubated with DMSO (as the control) and TF3 (160 *μ*M) for 24 h. MMP was detected by using a JC-1 assay kit (Beyotime, China). Following the manufacturer's protocol, the samples were stained with Hoechst 33342 dye solution and the JC-1 probe protected from light. The fluorescence signal was observed and captured under a fluorescence microscope (Nikon, Japan).

### 2.6. ROS Assay

The different OS cell lines were plated in 6-well cell culture plates at 1 × 10^5^ cells/well and 96-well cell culture plates at 3 × 10^3^ cells/well and incubated overnight to allow adherence. ROS levels were determined using the probe DCFH-DA (Beyotime, China). OS cells pretreated with DMSO (as the control) and OS cells pretreated with different concentrations of TF3 were incubated with the probe DCFH-DA following the manufacturer's protocol. Then, ROS generation of the cells in 6-well cell culture plates was visualized and estimated by fluorescence microscopy (Nikon, Japan), and the fluorescence intensity of the cells in 96-well cell culture plates was detected immediately at Ex/Em = 488/525 nm by a fluorescence reader (Thermo Fisher, Massachusetts, USA).

### 2.7. Measurement of MDA

MG63 and HOS cells were plated on 6-well cell culture plates at 1 × 10^5^ cells/well and incubated overnight to allow adherence. An MDA assay kit (Beyotime, China) was used to detect MDA content. The following day, OS cells pretreated with DMSO (as the control) and OS cells pretreated with different concentrations of TF3 were collected and lysed by using ice-cold RIPA lysis buffer (Beyotime, China). After centrifugation, the protein concentration of the samples was tested using a BCA Protein Assay Kit (Beyotime, China). The test solution was added to the supernatant and incubated at 100°C for 15 min. The samples were transferred to 96-well plates and measured via a multifunctional microplate reader (Thermo Fisher, Massachusetts, USA) at 532 nm, and the results were normalized to the total protein concentration.

### 2.8. Measurement of GSH

As recommended in the protocol, GSH levels were measured with a GSH assay kit (Solarbio, China). In brief, MG63 and HOS cells were plated at a density of 1 × 10^6^ cells/dish in 10 cm culture dishes and incubated overnight to allow adherence. The following day, OS cells pretreated with DMSO (as the control) or different concentrations of TF3 were washed with cold PBS and solubilized by sonication. After centrifugation, the supernatant was transferred to a 96-well plate, and the absorbance was measured by using a fluorescence microplate reader at 412 nm (Thermo Fisher, Massachusetts, USA). The data were normalized to the protein concentration.

### 2.9. Ferrous Iron (Fe^2+^) Detection

The intracellular Fe^2+^ levels were measured by using a FerroOrange probe (Dojindo, Japan) [13]. The different OS cell lines were plated in 6-well cell culture plates at 1 × 10^5^ cells/well and 96-well cell culture plates at 3 × 10^3^ cells/well and incubated overnight to allow adherence. According to the protocol, OS cells pretreated with DMSO (as the control) and various concentrations of TF3 for 24 h were incubated for 0.5 h at 37°C with FerroOrange (1 mM) dispersed in serum-free medium. The fluorescence was detected and visualized under a fluorescence microscope (Nikon, Japan), and the fluorescence intensity of the cells in 96-well cell culture plates was detected immediately at Ex/Em = 488/525 nm by a fluorescence reader (Thermo Fisher, Massachusetts, USA). The changes in Fe^2+^ are presented as the proportion of the fluorescence intensity value compared with that of the control.

### 2.10. Colony Formation Assay

The different OS cell lines were plated in 6-well cell culture plates at 500 cells/well, incubated overnight to allow adherence, and then incubated with DMSO (as the control) or different concentrations of TF3. After one week, the OS cells were fixed for 15 min with 4% paraformaldehyde and then washed twice with PBS. The fixed cells were stained for 15 min with 0.1% crystal violet and then washed twice with PBS. Microscopic observation was performed to define the viable cell colonies as those with at least 50 cells, and images were then obtained and quantified. The number of colonies was determined using the ImageJ software.

### 2.11. EdU Cell Proliferation Assay

The proliferation ability of different OS cell lines was detected with a BeyoClick™ EdU Cell Proliferation Kit with Alexa Fluor 555 (Beyotime, China). In brief, the different OS cell lines were plated in 12-well cell culture plates at 5 × 10^4^ cells/well and incubated overnight to allow adherence. The next day, the cells were incubated with DMSO (as the control) or different concentrations of TF3 (0, 40, 80, 120, or 160 *μ*M) for 24 h. According to the protocol, labelling was performed for 2 h with 10 *μ*M EdU, and immobilization was then performed for 30 min with PBS containing 4% paraformaldehyde. Afterward, the cells were permeabilized with permeable solution for 10 min and then washed three times with PBS. Thereafter, the cells were incubated with Click Additive Solution for 30 min in the dark. In the final step, the nuclei of the cells were counterstained with DNA-specific Hoechst 33342 dye in a light-free environment for 15 min. The Hoechst 33342 nuclear dye was removed, and the cells were washed three times with PBS. Representative staining images were captured with a fluorescence microscope (Nikon, Japan).

### 2.12. Morphological Apoptosis Analysis

MG63 and HOS cells were plated in 6-well plates at 1 × 10^5^ cells per well and cultured overnight for adherence. Cells treated with DMSO (as the control) or TF3 at various concentrations were studied for 24 h. Afterward, the culture medium was aspirated, and the cells were counterstained with Hoechst 33258 nuclear dye in a light-free environment for 15 min according to the manufacturer's protocol. Morphological changes in the nucleus were captured by using a fluorescence microscope (Nikon, Japan).

### 2.13. Apoptosis Analysis by Flow Cytometry

A propidium iodide (PI)/Annexin V-FITC kit was used to detect apoptosis in different OS cell lines. MG63 and HOS cells were plated in 6-well cell culture plates at 1 × 10^5^ cells/well and incubated to achieve adherence. After treatment with DMSO (as the control) or different concentrations of TF3, OS cells were collected. Flow cytometry samples were stained following the manufacturer's protocol. Apoptotic OS cells were identified, categorized, and quantified with a Beckman flow cytometer (CA, USA).

### 2.14. Cell Cycle Analysis

MG63 and HOS cells were plated in 6-well cell culture plates at 1 × 10^5^ cells/well and incubated to reach adherence. After treatment with DMSO (as the control) or different concentrations of TF3 for 24 h, OS cells were collected and then fixed for 16 h using chilled 70% ethanol at -20°C. The subsequent day, flow cytometry was performed. The samples were washed with cold PBS and treated with RNase to remove RNA after centrifugation (1500 rpm, 5 min, 22°C). Afterward, the OS cells were stained with PI for 10 min in the dark. Finally, cell cycle analysis was performed using a Beckman flow cytometer (CA, USA).

### 2.15. Western Blot Assay

The different OS cell lines were treated with different regimens for the indicated times. Then, the samples were lysed in RIPA lysis buffer (Beyotime, China) supplemented with PMSF (Beyotime, China) and phosphatase inhibitors (Beyotime, China). The supernatant was harvested after centrifugation at 13,000 × g for 10 min at 4°C. The protein levels were measured using a BCA protein assay kit (Beyotime, China). Proteins (30 *μ*g/lane) were separated by molecular weight via SDS–PAGE (EpiZyme, China) and transferred onto polyvinylidene difluoride membranes, which were blocked with either 5% BSA for 2 h or QuickBlock™ Blocking Buffer (Beyotime, China) at room temperature for 15-30 min. The membranes were then probed with the corresponding primary antibodies and appropriate secondary antibodies. The protein bands were visualized and analysed by using a hypersensitive electrochemiluminescence (ECL) detection kit and the ChemiDoc™ MP System (Bio-Rad, USA). Densitometric analyses of protein bands were performed by the ImageJ software.

### 2.16. OS Xenograft Model

The present study was conducted in accordance with the protocols approved by the Ethics Committee of the First Affiliated Hospital of Chongqing Medical University (No. 2022-K89). Five-week-old female BALB/c nude mice (18 g-20 g) were purchased from Hunan SJA Laboratory Animal Co., Ltd. (Hunan, China). All efforts were made to reduce animal suffering. All mice were housed under a specific pathogen-free environment with regular photoperiods and provided free access to food and water. The mice were acclimated for one week until study initiation. Then, HOS cells were harvested, resuspended in sterile PBS, and counted, and approximately 1 × 10^6^ HOS cells were immediately injected subcutaneously into the right flank of each mouse. Three days after inoculation, the mice were randomized into three groups of 5 mice per group and treated with different doses of TF3 (20, 40 mg/kg) or 0.05% DMSO (as the control) by gavage every day. The tumour sizes (length and width) were measured to calculate tumour volumes every 3 days, and the animals were deeply anaesthetized with isoflurane and then euthanized via CO_2_ asphyxiation and cervical dislocation after 22 days of treatment. Tumour tissues were harvested, measured, weighed, and then fixed with 4% paraformaldehyde. The hearts, livers, spleens, lungs, and kidneys were also collected, analysed, and fixed.

### 2.17. Histological Analysis

Harvested samples were dehydrated in a graded series of ethanol and xylene following fixation. Paraffin-embedded tissues were sectioned and further stained with haematoxylin and eosin (H&E) before histological images were observed and captured by light microscopy (Nikon, Japan).

For the measurement of ROS in xenograft tumour tissue, frozen sections of tumour tissue from the control or TF3-treated groups were incubated with the ROS fluorescent probe DHE (Beyotime, China). ROS measurement was performed according to the procedure of a ROS Assay Kit. The nuclei were counterstained with DAPI. Fluorescence images were observed and acquired with a fluorescence microscope (Nikon, Japan).

DAB-enhanced Perls' Prussian blue was used for histochemical determination of iron storage in xenograft tumour tissue. According to the protocol, the sections were placed into Prussian blue solution (Servicebio, Wuhan, China) and incubated for 30 min at room temperature. Thereafter, the DAB chromogenic solution (Servicebio, Wuhan, China) was added to enhance staining at room temperature. All sections were then rinsed, dehydrated, cover-slipped, and digitized via light microscopy (Nikon, Japan).

For immunohistochemistry, the sections were deparaffinized in xylene, sequentially rehydrated in ethanol, and quenched in 3% hydrogen peroxide. The sections were incubated with primary antibodies diluted in blocking buffer overnight at 4°C after blocking the slides for 60 min in Tris-buffered saline including 5% BSA along with 0.1% Tween 20 at room temperature. The antibodies used for immunohistochemistry targeted cleaved caspase-3 (1 : 2000 dilution, CST), Ki67 (1 : 500 dilution, CST), P21 (1 : 50 dilution, CST), JNK (1 : 200 dilution, Abcam), p-JNK (1 : 50 dilution, CST), FTH (1 : 100 dilution, Affbiotech), and GPX4 (1 : 1500 dilution, Abcam). Finally, the slides were visualized with DAB (Servicebio, China). TUNEL staining was performed by using a TUNEL Cell Apoptosis Detection Kit (G1501-100T, Servicebio, Wuhan, China) based on the manufacturer's protocol. Images were captured under a fluorescence microscope (Nikon, Japan).

### 2.18. Statistical Analysis

Statistical analyses were performed, and figures were generated with the GraphPad Prism 8.0 software. The data are displayed as the mean ± SD from at least three independent experiments, and all results were assessed for normality, unless otherwise noted. One-way analyses of variance (ANOVA) along with Tukey's test or Student's *t* test were used for statistical analysis of normally distributed data. A *P* value < 0.05 was considered to indicate significance.

## 3. Results

### 3.1. TF3 Impaired Viability and Caused Cell Death in Human OS Cells

To validate the biological effects of TF3 on human OS cells, human OS cells and the human normal osteoblastic cell line hFOB1.19 were tested for their viability after incubation with various concentrations of TF3 for different durations using a CCK-8 assay. As shown in Figures 1(b)–1(d), a concentration-dependent inhibitory effect of TF3 was observed on human OS cell lines, whereas no significant inhibitory effect was observed in hFOB1.19 cells, which indicated the selective cytotoxicity of TF3 to OS cells. Following treatment with TF3, the human OS cell lines underwent morphological changes. Light microscopy revealed that TF3 increased cell death and resulted in a rounded cell shape and shrunken cells as the concentration increased (Figure 1(e)).

### 3.2. TF3 Induced Ferroptosis, Altered Iron Metabolism, and Induced Oxidative Stress in Human OS Cells

**Figure 2:**
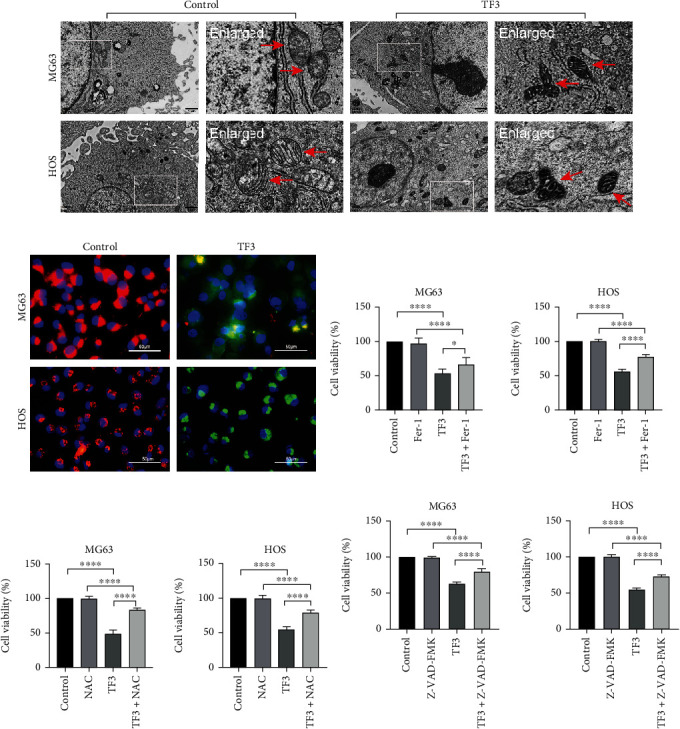
TF3 induced cell death in OS cell lines. OS cells were treated with 0.05% DMSO (vehicle control) or the indicated concentration (160 *μ*M) of TF3 for 24 h. (a) TEM was used to examine the ultrastructure of MG63 and HOS cells. The mitochondria of cells in the TF3 (160 *μ*M) group seemed to be shrunken, with dense membranes and diminished or absent cristae. (b) Merged images of JC-1 staining illustrating the differences in MMP between the TF3 (160 *μ*M) and control groups. The cells were also counterstained with Hoechst 33342 solution. Red fluorescence: polymers and normal MMP; green fluorescence: monomers and low MMP. (c–e) A CCK-8 assay was performed to measure the viability of HOS and MG-63 cells incubated with TF3 (160 *μ*M) for 24 h in the presence of 2 mM NAC, 2 *μ*M Fer-1, or 20 *μ*M z-VAD-FMK. The data are presented as the mean ± SD (*n* = 3). ^∗^*P* < 0.05, ^∗∗^*P* < 0.01, ^∗∗∗^*P* < 0.001, and ^∗∗∗∗^*P* < 0.0001 vs. the vehicle control group.

To examine TF3-induced ferroptosis in human OS cell lines, TEM was used to examine the subcellular structural changes in the treated cells. As shown in Figure 2(a), after treatment with TF3 (160 *μ*M) for 24 h, the mitochondria of MG63 and HOS cells exhibited typical morphological characteristics of ferroptotic cell death; specifically, they were rounded and shrunken, the membrane density was increased, and mitochondrial cristae were reduced in number or absent. In contrast, the mitochondria of the control group were elongated. Furthermore, JC-1-stained images revealed that the TF3 group samples (160 *μ*M for 24 h) appeared green, while the control group samples appeared red (Figure 2(b)). The mitochondrial membrane potential (MMP) was lower in MG63 and HOS cells treated with TF3 than in control cells. Finally, to clarify whether TF3 induced ferroptosis or apoptosis, the viability of MG63 and HOS cells treated with TF3 (160 *μ*M) in the presence of the antioxidant NAC (2 mM), the caspase inhibitor z-VAD-FMK (20 *μ*M), or the ferroptosis inhibitor Fer-1 (2 *μ*M) was assessed. The findings demonstrated that z-VAD-FMK, Fer-1, and NAC attenuated the cell death triggered by TF3 in OS cell lines (Figures 2(c)–2(e)). The inhibitors partially prevented human OS cells from dying, showing that TF3 treatment invoked ferroptosis and apoptosis in these cells.

**Figure 3:**
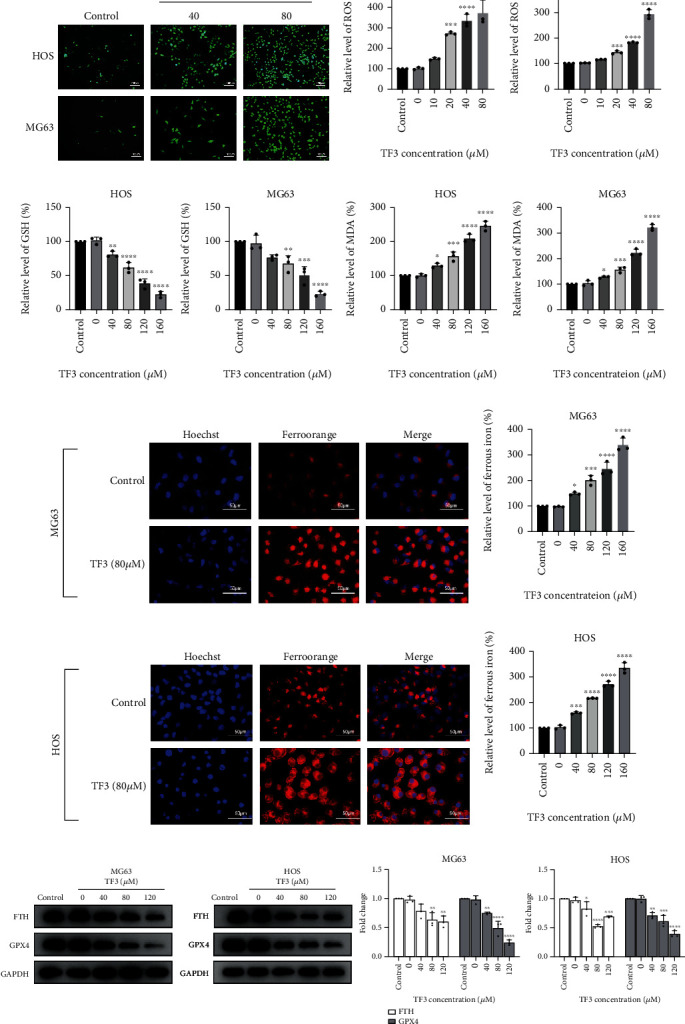
TF3 induced oxidative stress and altered iron metabolism in OS cell lines. OS cells were treated with 0.05% DMSO (vehicle control) or the indicated concentrations (0, 40, 80, 120, or 160 *μ*M) of TF3 for 24 h. (a) Representative fluorescence images of MG63 and HOS cells stained with DCFH-DA (green). (b) Statistical graphs of ROS levels detected with a fluorescence reader in MG63 and HOS cells stained with DCFH-DA (green). (c) Statistical graphs of GSH levels detected with a GSH assay kit in MG63 and HOS cells. (d) Statistical graphs of MDA levels detected with an MDA assay kit in MG63 and HOS cells. (e, g) Representative fluorescence images of cells stained with FerroOrange and Hoechst 33342 solution in the TF3 treatment and control groups. (f, h) Statistical graphs of Fe^2+^ levels detected by a fluorescence reader in MG-63 and HOS cells stained with FerroOrange solution. (i) Western blotting and statistical analyses of ferroptosis-related proteins, including FTH and GPX4, in MG63 and HOS cells. The data are presented as the mean ± SD (*n* = 3). ^∗^*P* < 0.05, ^∗∗^*P* < 0.01, ^∗∗∗^*P* < 0.001, and ^∗∗∗∗^*P* < 0.0001 vs. the vehicle control group.

To investigate whether TF3 induces oxidative stress in MG63 and HOS cells, the probe DCFH-DA was used to assess changes in cytosolic ROS levels in OS cell lines incubated with TF3 for 24 h. With increasing TF3 concentrations, ROS production gradually became evident in MG-63 and HOS cells (Figures 3(a) and 3(b)). Next, GSH and MDA levels were measured in human OS cells to determine whether ROS induced by TF3 had an oxidative stress effect, and the GSH content was significantly lower in the TF3-treated samples than in the control samples.

In addition, TF3 treatment of MG63 and HOS cells significantly increased the levels of MDA, which is a marker of lipid peroxidation (Figures 3(c) and 3(d)).

There are overlapping and precise regulatory mechanisms of iron metabolism, and altered iron metabolism can cause cancer development or cell death. To determine whether TF3 induces Fe^2+^ accumulation, we measured Fe^2+^ expression in MG63 and HOS cells after 24 h of treatment with different concentrations of TF3. Compared with the control treatment, TF3 increased Fe^2+^ concentrations in a concentration-dependent manner (Figures 3(e)–3(h)).

Finally, Western blotting was utilized to investigate the mechanism responsible for ferrous iron accumulation and ROS production caused by TF3. The concentration-dependent reductions in FTH and GPX4 expression caused by TF3 treatment are shown in Figure 3(i).

We concluded that TF3 damaged cellular antioxidant defence, reduced GPX4 expression, and activated the Fenton reaction induced by Fe^2+^ accumulation. Furthermore, ROS accumulation, lipid peroxidation, and oxidative stress were observed in MG63 and HOS cells.

### 3.3. TF3 Inhibited Proliferation and Triggered Cell Cycle Arrest in Human OS Cells

**Figure 4:**
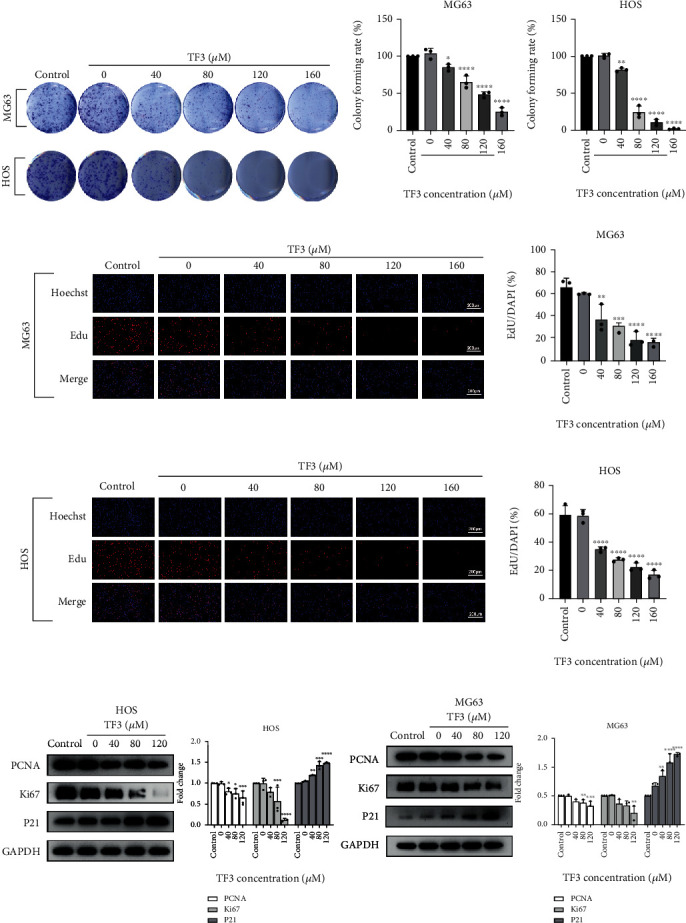
TF3 inhibited proliferation in OS cell lines. OS cells were treated with 0.05% DMSO (vehicle control) or the indicated concentrations (0, 40, 80, 120, or 160 *μ*M) of TF3 for the indicated times. (a) The colony formation ability of MG-63 and HOS cells was determined. (b) Quantitative analyses of the colonies in (a). (c, d) The proliferation of MG-63 and HOS cells was assessed through EdU staining and quantification. (e) Western blotting and statistical analyses of proliferation-related proteins, including Ki67, PCNA, and P21, in MG-63 and HOS cells. The data are presented as the mean ± SD (*n* = 3). ^∗^*P* < 0.05, ^∗∗^*P* < 0.01, ^∗∗∗^*P* < 0.001, and ^∗∗∗∗^*P* < 0.0001 vs. the vehicle control group.

**Figure 5:**
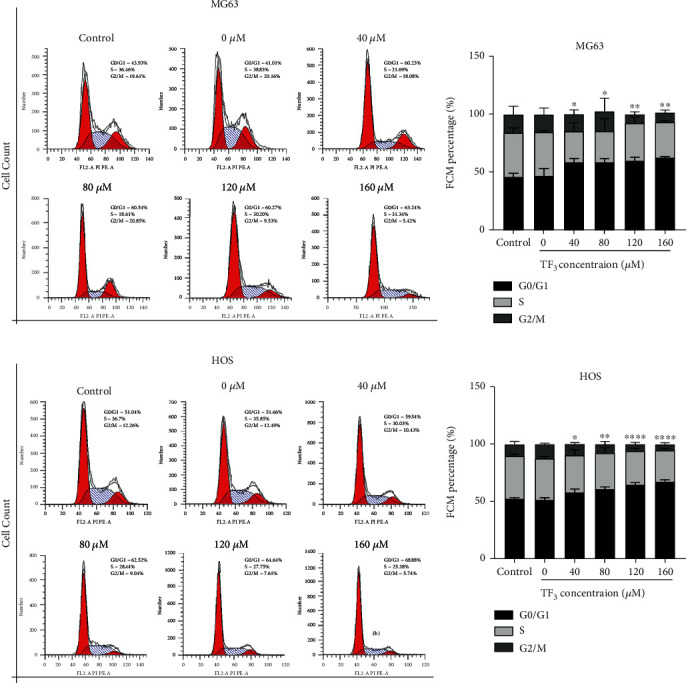
TF3 induced G0/G1 cell cycle arrest in OS cell lines. OS cells were treated with 0.05% DMSO (vehicle control) or the indicated concentrations (0, 40, 80, 120, or 160 *μ*M) of TF3 for 24 h. (a, b) Cell cycle analyses of MG-63 and HOS cells via flow cytometry and quantitative analyses of the cell cycle distributions. The data are presented as the mean ± SD (*n* = 3). ^∗^*P* < 0.05, ^∗∗^*P* < 0.01, ^∗∗∗^*P* < 0.001, and ^∗∗∗∗^*P* < 0.0001 vs. the vehicle control group.

Assays of colony formation and cell proliferation using EdU were conducted to determine the antiproliferative properties of TF3 in MG63 and HOS cells. TF3 treatment significantly reduced colony formation (Figures 4(a) and 4(b)). Additionally, as shown in Figures 4(c) and 4(d), the TF3-treated groups exhibited reduced numbers of total cells and EdU-positive cells, and the proportion of EdU-positive cells was significantly lower in the TF3-treated groups than in the control group. Furthermore, Western blotting revealed that the proliferation-related proteins PCNA and Ki67 were significantly downregulated, while the cell cycle-associated protein P21 was clearly upregulated, in the TF3 treatment groups in a concentration-dependent manner (Figure 4(e)). Finally, the role of TF3 in cell cycle progression was analysed to confirm the link between growth inhibition and the cell cycle. Compared with the control group, the groups of human OS cells treated with various concentrations of TF3 exhibited declines in the G2/M peak as well as an accumulation of cells in G0/G1 phase after 24 h of treatment with various concentrations of TF3 (Figures 5(a) and 5(b)). Taken together, these findings indicated that TF3 suppressed the proliferation of human OS cell lines in a manner regulated by proteins associated with proliferation and the cell cycle.

### 3.4. TF3 Induced Apoptosis in Human OS Cells

**Figure 6:**
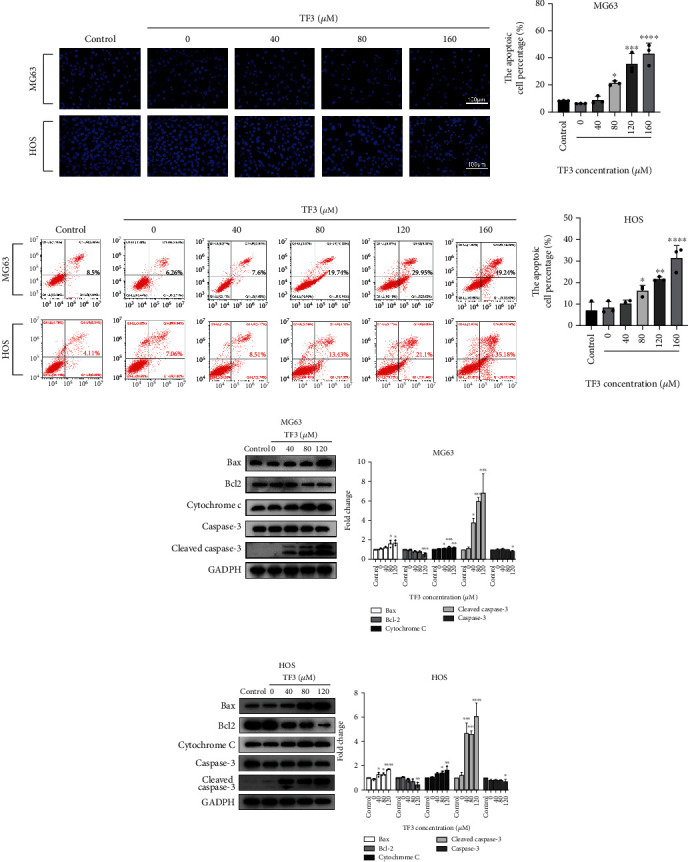
TF3 promoted apoptosis in OS cell lines. OS cells were treated with 0.05% DMSO (vehicle control) or the indicated concentrations (0, 40, 80, 120, or 160 *μ*M) of TF3 for 24 h. (a) Hoechst 33258 staining assay of MG-63 and HOS cells. (b–d) Flow cytometry analyses of cell apoptosis in MG-63 and HOS cells. (e, f) Western blotting and statistical analyses of apoptosis-related proteins, including Bax, Bcl-2, cytochrome C, caspase-3, and cleaved caspase-3, in MG-63 and HOS cells. The data are presented as the mean ± SD (*n* = 3). ^∗^*P* < 0.05, ^∗∗^*P* < 0.01, ^∗∗∗^*P* < 0.001, and ^∗∗∗∗^*P* < 0.0001 vs. the vehicle control group.

As tumorigenesis is closely linked to apoptosis, we explored how TF3 influences apoptosis in OS cells. After TF3 treatment, typical morphological changes associated with apoptosis, such as nuclear size reduction, nuclear fragmentation, and chromatin condensation, were observed by staining with Hoechst 33258 in OS cell lines (Figure 6(a)). Similarly, quantitative analysis of apoptosis based on flow cytometry revealed that TF3 may have significantly induced apoptosis in treated MG63 and HOS cells compared with control cells (Figures 6(b)–6(d)). Western blotting was performed to identify apoptosis-related proteins involved in TF3-induced apoptosis in human OS cells. Bcl-2 and caspase-3 protein levels were significantly downregulated, while Bax, cytochrome C, and cleaved caspase-3 protein levels were all significantly upregulated (Figures 6(e) and 6(f)). Therefore, the above data suggest that TF3 is a proapoptotic factor in OS cells.

### 3.5. TF3 Induced Activation of the MAPK Pathway by Triggering ROS Generation

**Figure 7:**
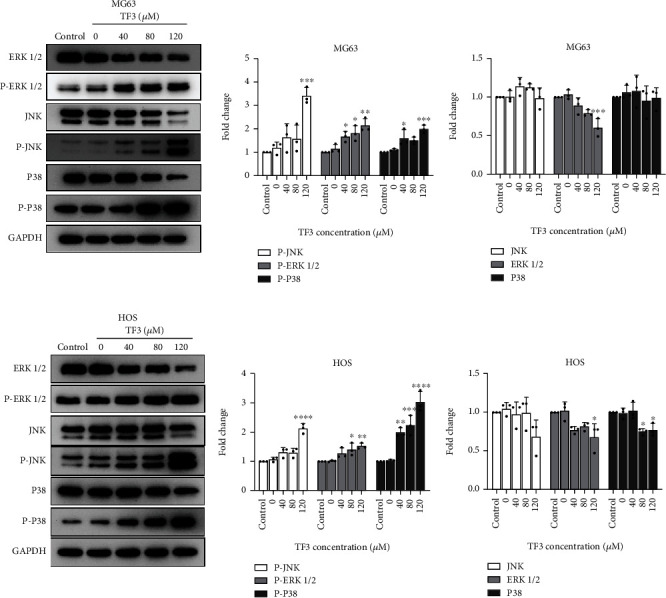
TF3 activated the MAPK pathway in OS cell lines. OS cells were treated with 0.05% DMSO (vehicle control) or the indicated concentrations (0, 40, 80, and 120 *μ*M) of TF3 for 24 h. (a, b) Western blotting and statistical analyses of MAPK signalling pathway-related proteins, including p-JNK, p-P38, p-ERK, JNK, P38, and ERK, in MG63 and HOS cells. The data are presented as the mean ± SD (*n* = 3). ^∗^*P* < 0.05, ^∗∗^*P* < 0.01, ^∗∗∗^*P* < 0.001, and ^∗∗∗∗^*P* < 0.0001 vs. the vehicle control group.

**Figure 8:**
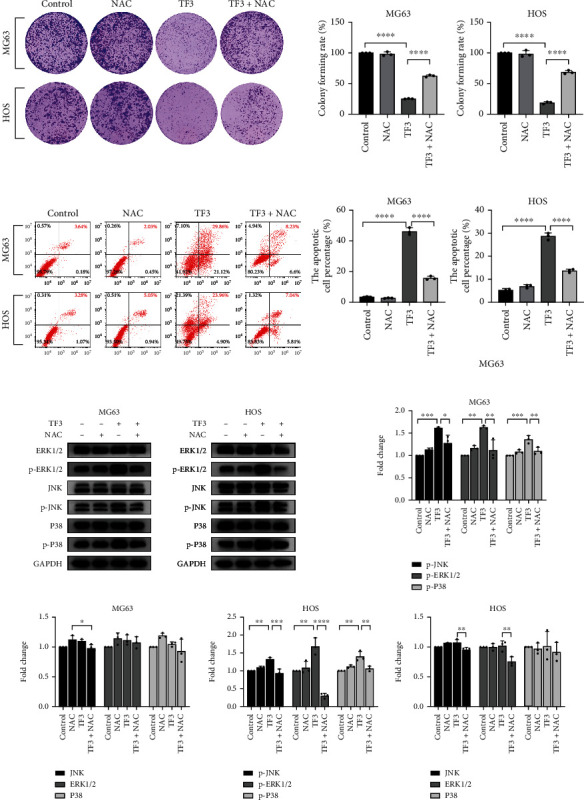
TF3 induced cell death in OS cell lines via ROS accumulation. (a) Colony formation assay of MG63 and HOS cells treated with 160 *μ*M TF3 with or without 2 mM NAC for 24 h and statistical analysis of the colony formation assay results. (b) Flow cytometry of MG63 and HOS cells treated with 160 *μ*M TF3 with or without 2 mM NAC for 24 h and quantitative analysis of the flow cytometry results. (c) Western blotting and statistical analyses of MAPK signalling pathway-related proteins, including p-JNK, p-P38, p-ERK, JNK, P38, and ERK, in MG63 and HOS cells treated with 160 *μ*M TF3 with or without 2 mM NAC for 24 h. The data are presented as the mean ± SD (*n* = 3). ^∗^*P* < 0.05, ^∗∗^*P* < 0.01, ^∗∗∗^*P* < 0.001, and ^∗∗∗∗^*P* < 0.0001 vs. the vehicle control group.

The MAPK pathway plays an important role in inducing either apoptosis or cell survival in OS cell lines [14, 15]. Thus, we sought to detect the functions of TF3 in MG63 and HOS cells. Compared with the control, TF3 gradually induced JNK, p38, and ERK activity in human OS cells in a dose-dependent manner, as shown in Figures 7(a) and 7(b). The phosphorylation levels of JNK, p38, and ERK were most significantly altered in the 120 *μ*M group. Human OS cells were then cotreated with NAC in a series of procedures to verify whether ROS generation was an upstream signal in TF3-induced growth inhibition and cell death. As shown in Figure 8(a), the proliferation inhibition caused by TF3 was significantly alleviated when MG63 and HOS cells were cotreated with NAC, as indicated by a colony formation assay. The cells pretreated with NAC exhibited significantly less TF3-induced apoptosis than the cells treated with TF3 alone (Figure 8(b)). Since TF3 triggered the MAPK pathway, we investigated whether ROS are involved in MAPK phosphorylation. Western blotting revealed that TF3-induced JNK, P38, and ERK phosphorylation was obviously inhibited by pretreatment with NAC (Figure 8(c)), which suggested that the ROS generated by TF3 in human OS cells were crucial for inhibiting cell proliferation, triggering cell death, and activating the MAPK signalling pathway.

### 3.6. TF3 Prevented the Growth of OS in Xenograft Tumours

**Figure 9:**
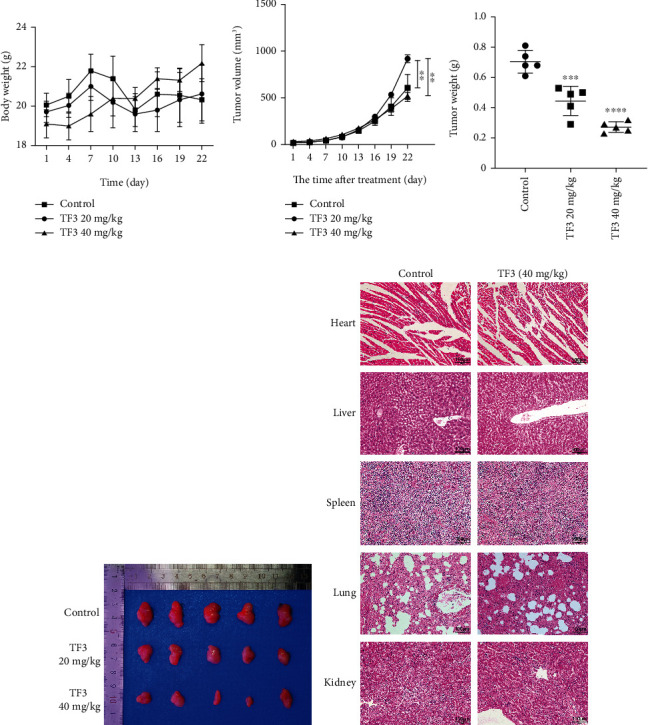
TF3 suppressed tumour growth in a xenograft model. (a) Body weights were measured every 3 days after TF3 treatment. (b) Tumour volume was recorded every 3 days after TF3 treatment. (c) Tumour weights were measured after all mice were killed. (d) Representative images of tumours in the different treatment groups. (e) The histological characteristics of major organs were assessed using H&E staining. The data are presented as the mean ± SD (*n* = 5). ^∗^*P* < 0.05, ^∗∗^*P* < 0.01, ^∗∗∗^*P* < 0.001, and ^∗∗∗∗^*P* < 0.0001 vs. the vehicle control group.

**Figure 10:**
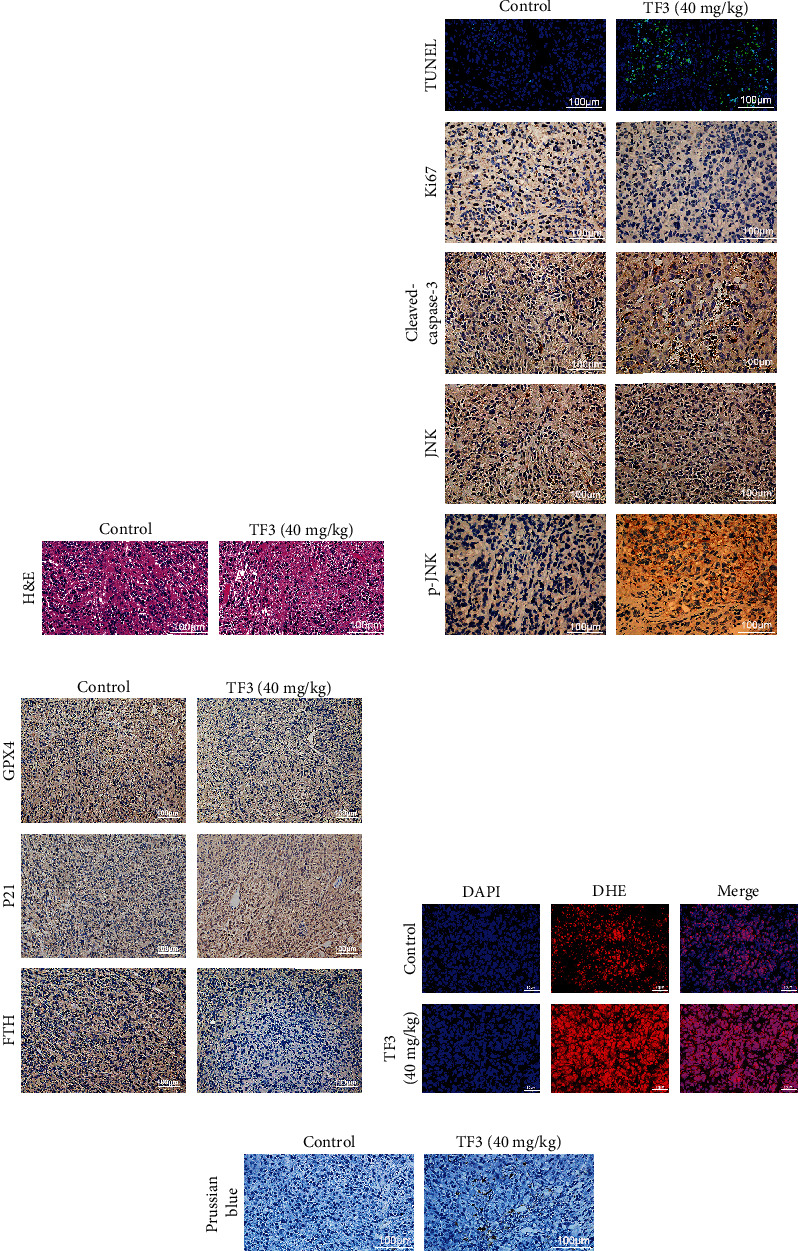
TF3 induced tumour cell death in a xenograft model. (a) The effects of TF3 on xenograft tumours were assessed via H&E staining. (b, c) The expression of TUNEL, Ki67, cleaved caspase-3, JNK, p-JNK, GPX4, P21, and FTH was observed by immunohistochemistry. (d) TF3 increased ROS generation in tumour tissue. Representative images are shown (red fluorescence: DHE and ROS; blue fluorescence (DAPI): nucleus). (e) DAB-enhanced Perls' iron staining of the tumour.

To assess the antitumour effect of TF3 in vivo, a xenograft OS model was constructed by injecting HOS cells subcutaneously into BALB/c mice. TF3 significantly reduced OS growth in the 20 and 40 mg/kg treatment groups but did not significantly affect body weight (Figures 9(a)–9(d)). H&E staining revealed no obvious pathological changes in the main organs in the TF3 treatment group (Figure 9(e)), while OS cells treated with TF3 exhibited cell shrinkage, nuclear condensation, and lysis (Figure 10(a)), which are typical signs of apoptosis. ROS and iron levels were markedly higher in tumour tissue from the TF3 group than in tumour tissue from the control group (Figures 10(d) and 10(e)), which was consistent with the in vitro results. Immunohistochemistry indicated that the number of TUNEL-positive cells and the protein expression levels of cleaved caspase-3, P21, and p-JNK were increased, whereas the expression levels of Ki67, GPX4, FTH, and JNK were decreased (Figures 10(b) and 10(c)). Together, all of these data indicated that OS cell growth in vivo was inhibited by TF3 at a safe dose.

## 4. Discussion

**Figure 11:**
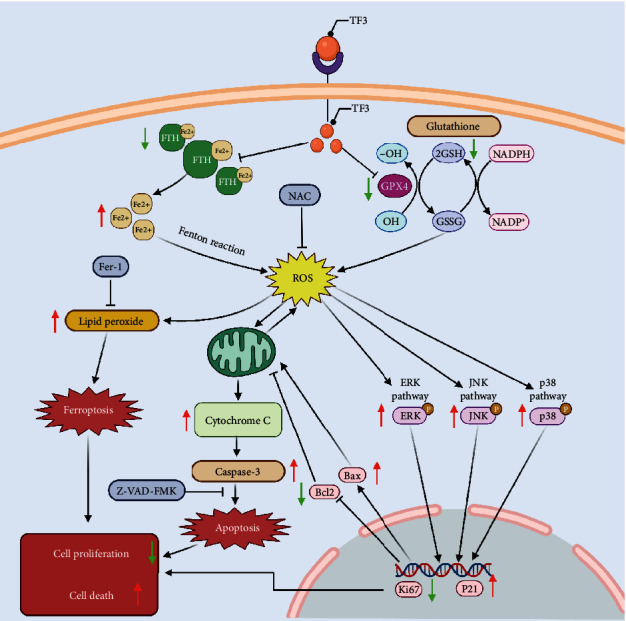
Schematic of the potential mechanism of TF3-induced proliferation inhibition, apoptosis, and ferroptosis.

TF3, an important polyphenolic compound in black tea, has demonstrable anticancer benefits. TF3 inhibits several human cancers, including gastric cancer, liver cancer, breast cancer, and lung cancer [16–18]. In the present study, human OS cells were damaged by TF3, which caused G0/G1 cell cycle arrest, inhibited proliferation, and induced two main types of programmed cell death. These effects were closely related to labile iron overload, GSH reduction, generation of ROS, and activation of the MAPK pathway (Figure 11).

Tumour cells display specific biological characteristics, such as uncontrolled proliferation and apoptosis blockade [19]. Inhibition of proliferation and activation of apoptosis may be effective ways to treat OS. TF3 has been shown to suppress cell proliferation while promoting cell death in various cancer cell lines [9]. In the current study, TF3 activated caspase-3, Bax, and cytochrome C to induce apoptosis in OS cells while inhibiting proliferation and arresting cell cycle progression at G0/G1 phase by decreasing Ki67 and PCNA levels. Mitochondria are an integral part of the intrinsic pathway of apoptosis, and loss of MMP induces apoptosis by causing cytochrome c release [20]. The current study demonstrated a significant decrease in MMP following exposure to TF3, which induced the release of cytochrome c. Notably, the cytotoxic effect of TF3 on OS cells was partially reduced when apoptosis was blocked with z-VAD-FMK, a pan-caspase inhibitor. In addition, the impairment of viability after TF3 treatment was also attenuated when a ferroptosis inhibitor (Fer-1) was administered. Therefore, we conclude that TF3 can trigger both apoptosis and other forms of programmed cell death.

Ferroptosis is a newly identified iron-dependent form of nonapoptotic cell death and is characterized by an overload of lipid peroxidation products and lethal ROS [21]. Several studies have suggested that the induction of ferroptosis has great potential for treating OS [22]. TF3 induced GSH reduction, mitochondrial shrinkage, labile iron accumulation, GPX4 suppression, and lipid peroxidation in the current study. Therefore, our results strongly suggest that TF3 initiates ferroptosis in the MG63 and HOS cell lines.

Ferritin is a key component of iron metabolism and is essential for cell growth and proliferation in mammals. The heavy chain catalyses the first step in iron storage, which is very important for the conversion of ferrous (Fe2^+^) to ferric (Fe3^+^) ions [23, 24]. However, iron overload or iron metabolism disorders can produce toxic ROS via the Fenton reaction, which can damage cells [25]. Our results demonstrated that TF3 downregulated FTH expression in vivo and in vitro to promote the release of Fe^2+^ and the generation of ROS that are involved in ferroptosis progression [26].

ROS act as signalling molecules that are crucial for redox homeostasis, but they are also toxic and closely related to various pathologies [27, 28]. Mitochondria produce and absorb ROS, and damaged mitochondria emit more ROS than normal mitochondria [29, 30]. Excessive ROS have been shown to mediate significant antitumour effects in OS cells in some studies [31–33]. A decrease in the activity of GPX4 leads to a reduction in GSH synthesis, resulting in cellular antioxidant depletion, ROS accumulation, and ferroptosis [34]. Shi et al. [35] found that tirapazamine causes ferroptosis by downregulating the expression of GPX4 in OS cells. Luo et al. [36] also reported that bavachin triggers ferroptosis by inhibiting GPX4 in OS cells. Similarly, in the present study, TF3 treatment increased the generation of cellular ROS and the levels of MDA, while it induced a reduction in GSH and downregulated the expression of GPX4, eventually resulting in ferroptosis.

Additionally, ROS are involved in the MAPK pathway, which modulates signal transduction and is associated with many biological functions, such as proliferation, survival, and apoptosis [37–39]. MAPK signalling is a double-edged pathway that can promote tumour progression and tumour cell death in vivo and in vitro [40–43]. The same phenomenon is also observed in osteosarcoma [44–49]. Nevertheless, some chemopreventive agents activate MAPK signalling, leading to significant antiosteosarcoma effects [50, 51]. ROS overload can provoke mitochondrial dysfunction, promote a decrease in MMP, and increase the release of cytochrome C, eventually resulting in apoptosis [52]. The MAPK signalling pathway activated by ROS plays key roles in both apoptosis and ferroptosis of OS cells in vivo and in vitro [14, 33]. In the present experiments, we found that TF3 increased the generation of cellular ROS, induced both apoptosis and ferroptosis in MG63 and HOS cells, and activated the MAPK pathway. NAC attenuated the effects of TF3 on proliferation, survival, and the MAPK pathway. In summary, these results indicate that ROS-dependent MAPK signalling plays important roles in TF3-induced apoptosis and ferroptosis.

The anticancer potential of theaflavins against tumorigenesis and tumour development has been demonstrated in some animal models [53–56]. However, there has been no evidence that TF3 affects OS in animal models. We confirmed the effects of TF3 in xenograft models. Intragastric TF3 administration resulted in reduced systemic toxicity and exerted significant antitumour effects in OS xenograft models by altering iron metabolism and iron-dependent ROS accumulation, activating MAPKs, and inducing cell death. The present findings reveal that TF3 is a safe and promising potential agent for the treatment of OS.

## 5. Conclusions

In conclusion, a ROS-inducing strategy is a potential therapeutic avenue to treat OS. The present results indicate, for the first time, that TF3 promotes ferroptosis and apoptosis in human OS by triggering oxidative stress, inducing mitochondrial dysfunction and activating MAPK signalling pathways. Although the cross talk and relationships among the signalling pathways activated by TF3 are still unclear and require deeper investigation, TF3 appears to be a promising treatment for human OS.

## Data Availability

The datasets used and analysed during the current study are available from the corresponding author on reasonable request.
